# Author Correction: Unexpected limitation of tropical cyclone genesis by subsurface tropical central-north Pacific during El Niño

**DOI:** 10.1038/s41467-023-37523-8

**Published:** 2023-03-27

**Authors:** Cong Gao, Lei Zhou, Chunzai Wang, I.-I. Lin, Raghu Murtugudde

**Affiliations:** 1 School of Oceanography, Shanghai Jiao Tong University, Shanghai, China; 2 Southern Marine Science and Engineering Guangdong Laboratory (Zhuhai), Zhuhai, China; 3 State Key Laboratory of Tropical Oceanography, South China Sea Institute of Oceanology, Chinese Academy of Sciences, Guangzhou, China; 4 Department of Atmospheric Sciences, National Taiwan University, Taipei, Taiwan; 5 Department of Atmospheric and Oceanic Science, University of Maryland, College Park, MD USA; 6 Indian Institute of Technology, Bombay, Mumbai, India

**Keywords:** Physical oceanography, Atmospheric dynamics, Projection and prediction

Correction to: *Nature Communications* 10.1038/s41467-022-35530-9, published online 14 December 2022

The original version of this Article contained an error in the Results, subsection “Negative correlation between *D*_26_ and SST in central-north Pacific”, which incorrectly read ‘The conspicuous negative correlations between spatial mean wind stress curl and *D*_26_ are statistically significant as illustrated in Fig. 5c, d.’ The correct version adds ‘Ekman upwelling velocity induced by’ after ‘spatial mean’. This has been corrected in both the PDF and HTML versions of the Article.

The original version of this Article contained an error in the caption of Figure 5c, which incorrectly read ‘The scatter plot of regional mean wind stress curl anomalies’. The correct version replaces sentence with ‘The scatter plot of regional mean Ekman upwelling velocity anomalies’. This has been corrected in both the PDF and HTML versions of the Article.

The original version of this Article contained an error in Equation (3). The equation was given with an extra minus sign, and incorrectly read:

$${w}_{e}=-\frac{1}{\rho }\left(\frac{\partial {M}_{x}}{\partial x}+\frac{\partial {M}_{y}}{\partial y}\right)\ldots (3)$$

The correct form of Equation (3) is:

$${w}_{e}=\frac{1}{\rho }\left(\frac{\partial {M}_{x}}{\partial x}+\frac{\partial {M}_{y}}{\partial y}\right)\ldots (3).$$

This has been corrected in both the PDF and HTML versions of the Article.

