# Supplementary material for: Unexpected limitation of tropical cyclone genesis by subsurface tropical central-north Pacific during El Niño
Source: Nat Commun. 2022 Dec 14;13:7746. doi: 10.1038/s41467-022-35530-9 (PMC9751289; doi:10.1038/s41467-022-35530-9)
Supplement: Supplementary file 1 — Supplementary Information [file 41467_2022_35530_MOESM1_ESM.docx]

Unexpected Limitation of Tropical Cyclone Genesis by Subsurface Tropical Central-North Pacific during El Niño

**AUTHORS**

Cong Gao^1^, Lei Zhou^1, 2^*, Chunzai Wang^3^, I.-I. Lin^4^, Raghu Murtugudde^5, 6^

^1^School of Oceanography, Shanghai Jiao Tong University; Shanghai, China.

^2^Southern Marine Science and Engineering Guangdong Laboratory (Zhuhai); Zhuhai, China.

^3^State Key Laboratory of Tropical Oceanography, South China Sea Institute of Oceanology, Chinese Academy of Sciences; Guangzhou, China.

^4^Department of Atmospheric Sciences, National Taiwan University; Taipei, Taiwan.

^5^Department of Atmospheric and Oceanic Science, University of Maryland; College Park, Maryland, USA.

^6^Indian Institute of Technology – Bombay; Mumbai, India.

*Corresponding author: Lei Zhou (email: [zhoulei1588@sjtu.edu.cn](mailto:zhoulei1588@sjtu.edu.cn))

This PDF contains Supplementary Figures 1-9 and Supplementary Tables 1-2.


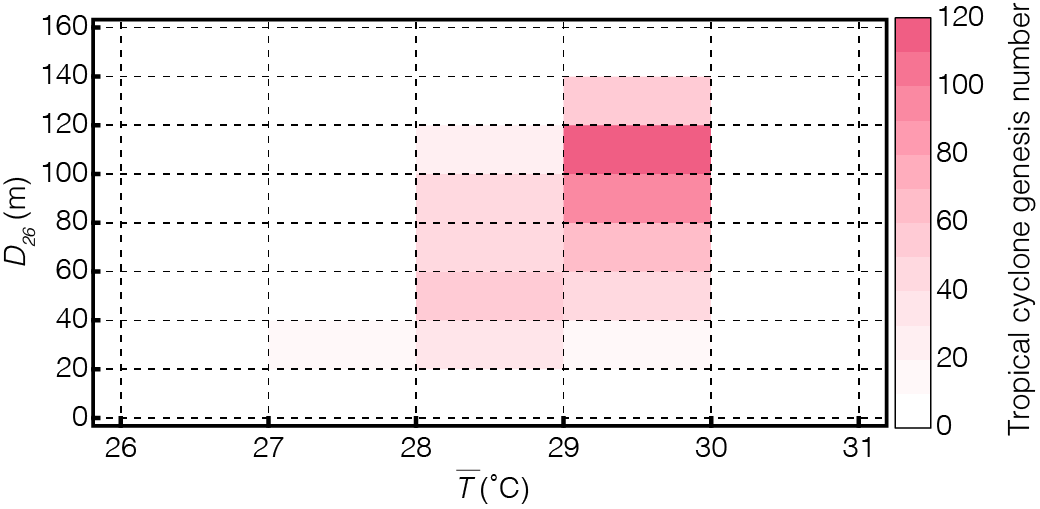


**Supplementary Fig. S1 | Joint distribution of tropical cyclone genesis numbers with respect to the mean temperature in the upper mixed layer (**$\bar{\boldsymbol{T}}\boldsymbol{)}$ **and 26ºC isotherm depth (**$\boldsymbol{D}_{\boldsymbol{26}}$**).** More tropical cyclones are formed when $D_{26}$ is deeper given the same $\bar{T}$. The conclusion stays valid even if the bin size of $\bar{T}$ is reduced.


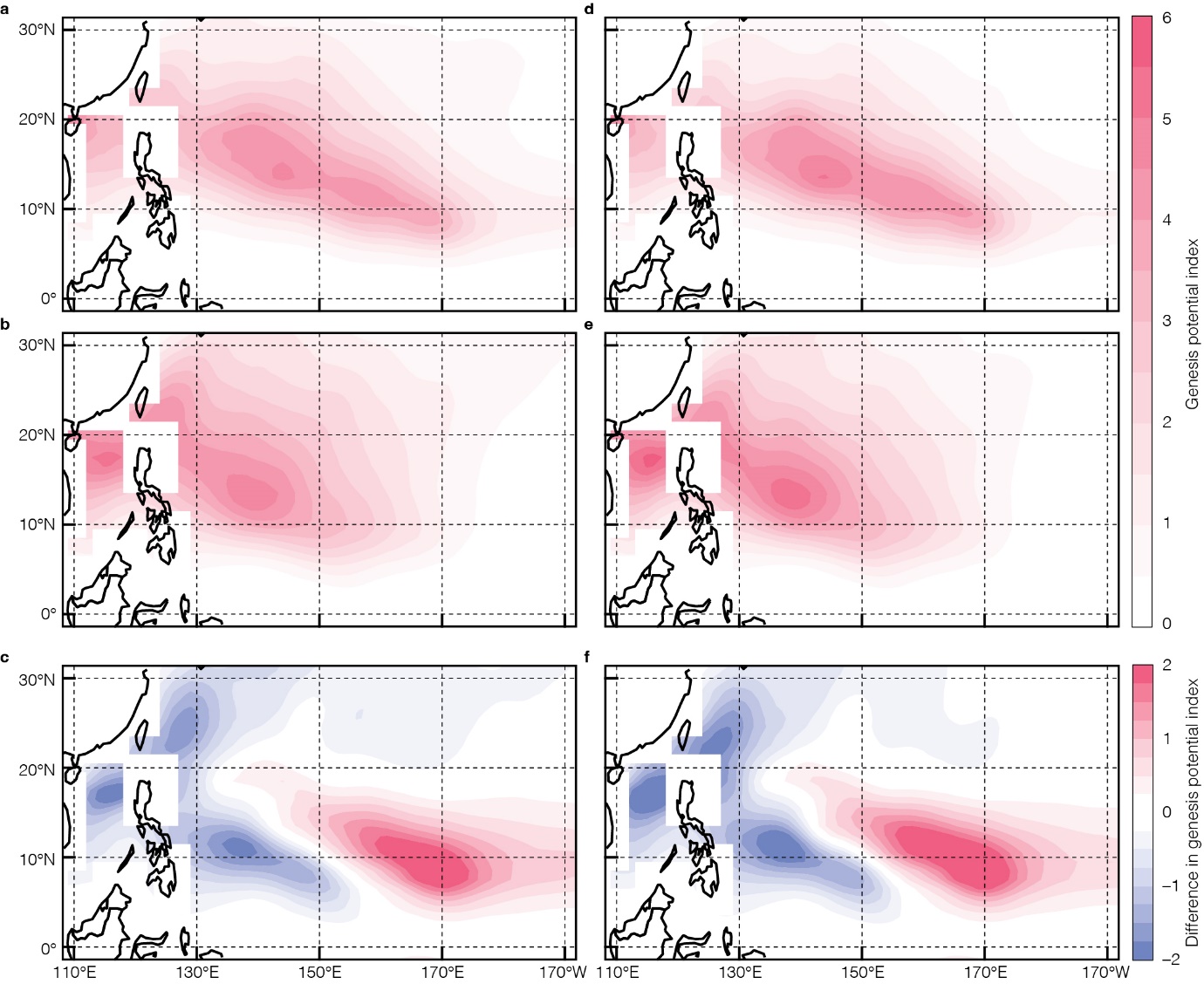


**Supplementary Fig. S2 |** **Tropical cyclone genesis numbers during El Niño and La Niña and their differences.** Same as Fig. 2a to 2c, but **a** to **c** are obtained using the genesis potential index (GPI) created by (28), and **d** to **f** are obtained with the GPI created by (29).


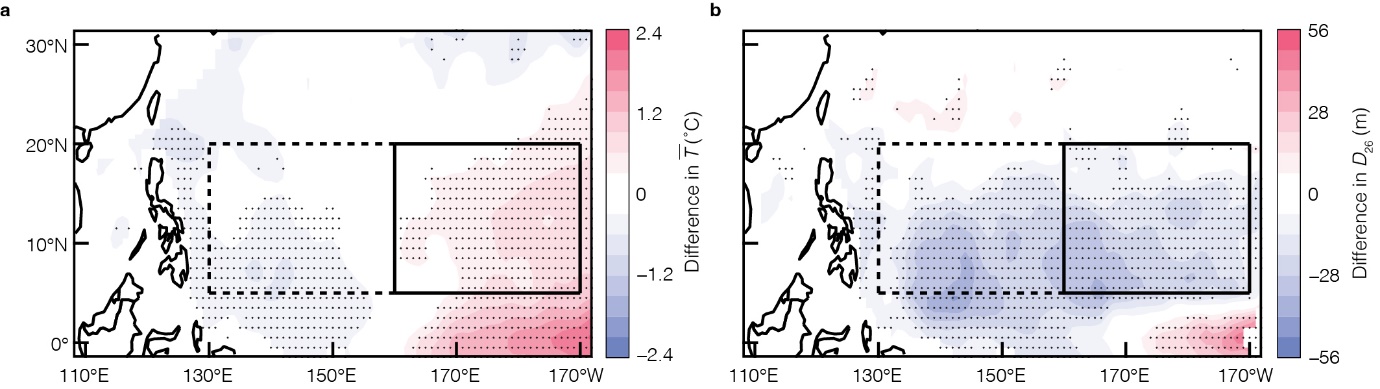


**Supplementary Fig. S3 |** **Differences of the upper mixed layer (**$\bar{\boldsymbol{T}}\boldsymbol{)}$ **and 26ºC isotherm depth (**$\boldsymbol{D}_{\boldsymbol{26}}$**) between the two phases of ENSO based on BOA_Argo data.** **a** is for $\bar{T}$ and **b** is for $D_{26}$. The temporal coverage is from 2004 to 2019.


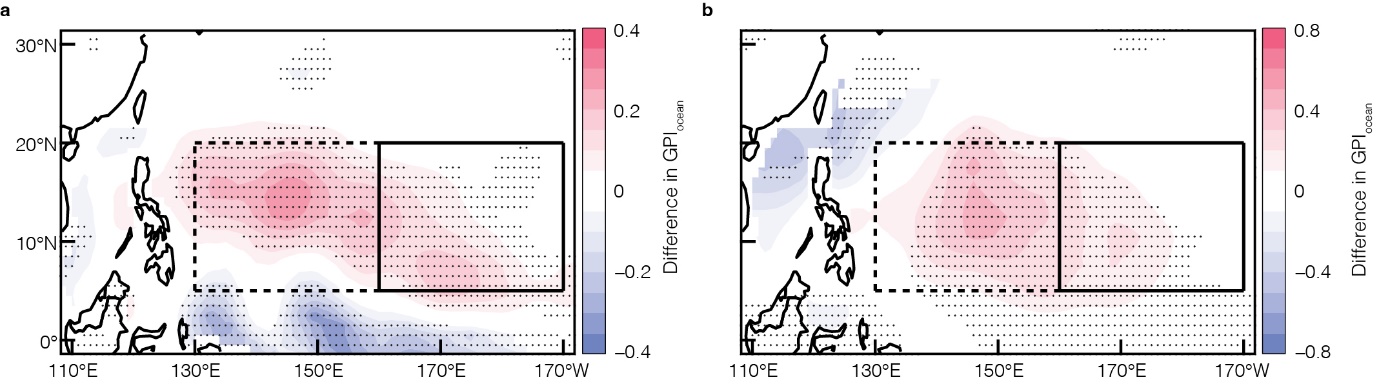


**Supplementary Fig. S4 | The differences of tropical cyclone genesis potential index (GPI) due to two atmospheric variables**. **a** The impacts of absolute vorticity at 1000 hPa ($\eta_{1000}$) on tropical cyclone genesis during El Niño. **b** Same as **a** but for the net long wave radiation at the sea surface ($F$).


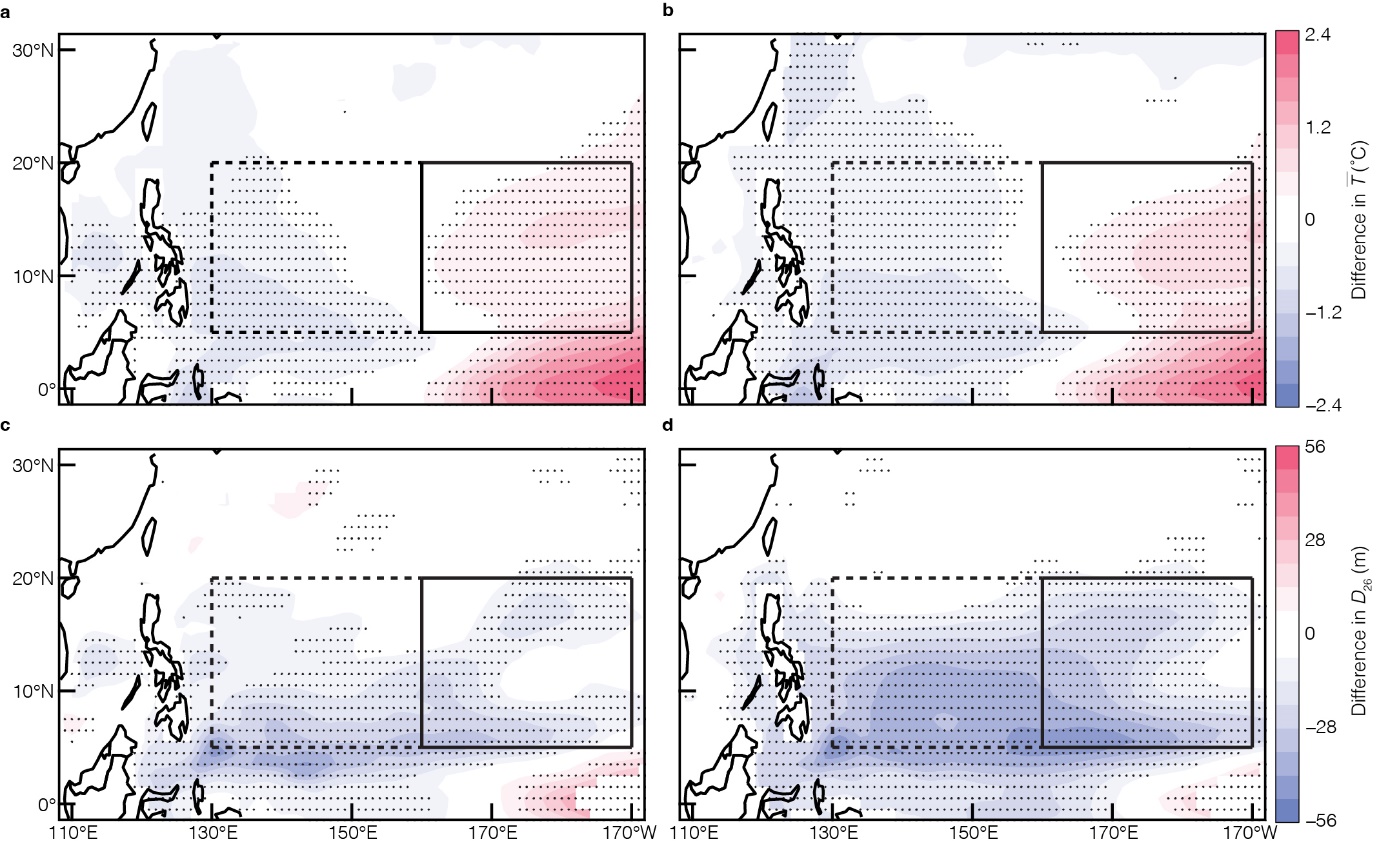


**Supplementary Fig. S5 |** **The differences of the upper mixed layer (**$\bar{\boldsymbol{T}}\boldsymbol{)}$ **and 26ºC isotherm depth (**$\boldsymbol{D}_{\boldsymbol{26}}$**) are independent of the types of El Niño. a** The difference of $\bar{T}$ between Central-Pacific (CP) El Niño and La Niña. **b** The difference of $\bar{T}$ between Eastern-Pacific (EP) El Niño and La Niña. **c** Same as **a** but for $D_{26}$. **d** Same as **b** but for $D_{26}$.


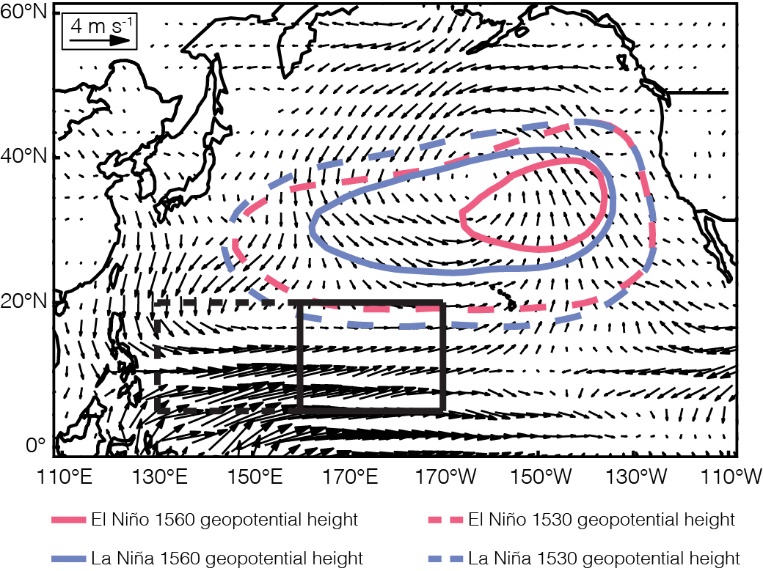


**Supplementary Fig. S6 |** **Weakening of the North Pacific Subtropical High during El Niño.** The red solid and dashed lines are the contours of 1560 and 1530 geopotential height (in meters) for El Niño, respectively. The blue lines are the same as the red lines but for La Niña. The vectors delineate 10-m wind anomalies just as in Fig. 5a.


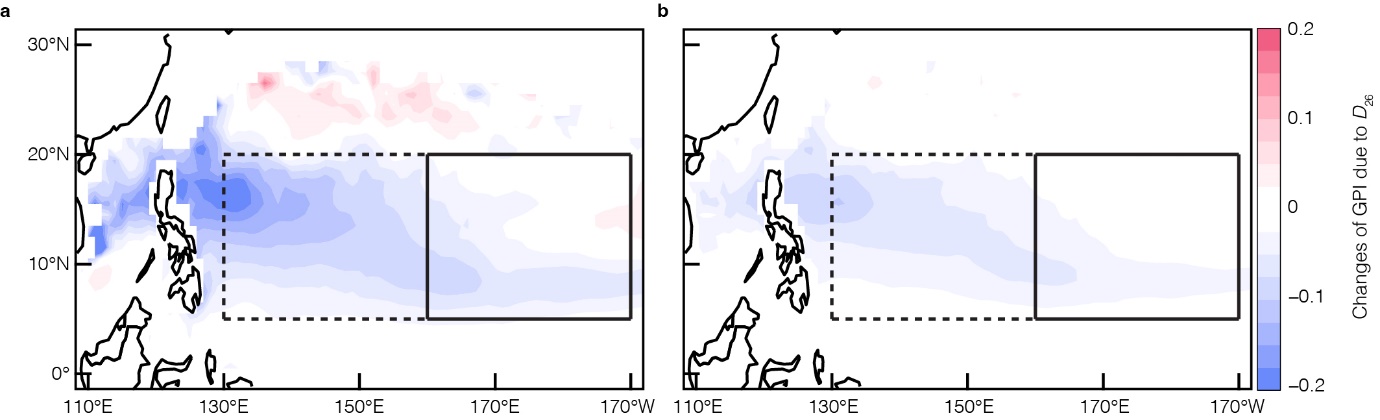


**Supplementary Fig. S7** | **The impacts of 26ºC isotherm depth (**$\boldsymbol{D}_{\boldsymbol{26}}$**) on** **tropical** **cyclone** **genesis potential index (GPI) derived from** **10 HighResMIP models.** **a** The impact based on GPI_ocean_. **b** The impact based on GPI_atm-ocean_.


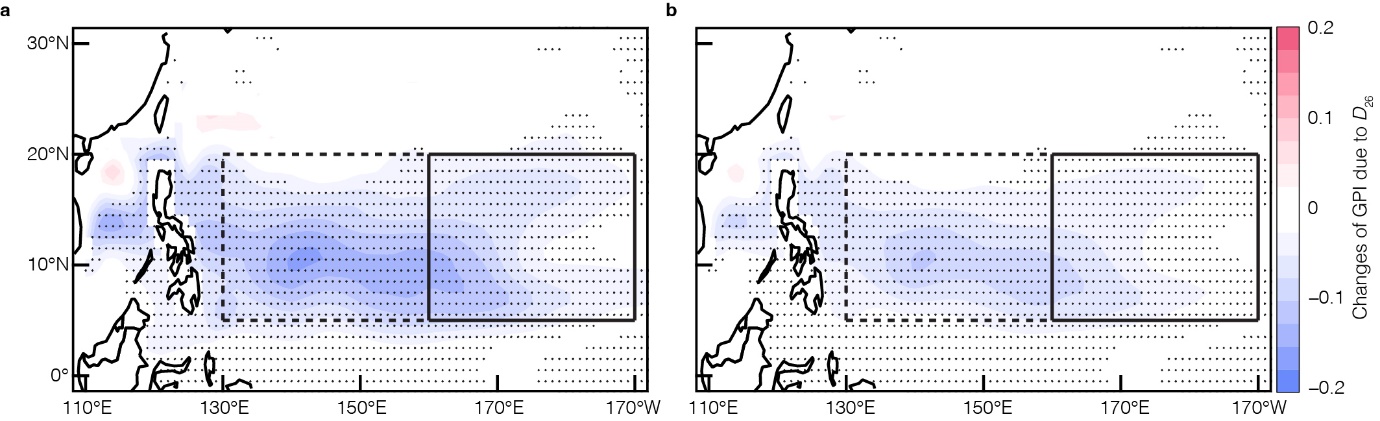


**Supplementary Fig. S8** | **The impacts of 26ºC isotherm depth (**$\boldsymbol{D}_{\boldsymbol{26}}$**) on** **tropical cyclone genesis potential index (GPI)** **derived from observations.** **a** The impact based on GPI_ocean_. **b** The impact based on GPI_atm-ocean_. **a** is the replication of Fig. 3d for the convenience of comparison with **b**.

**
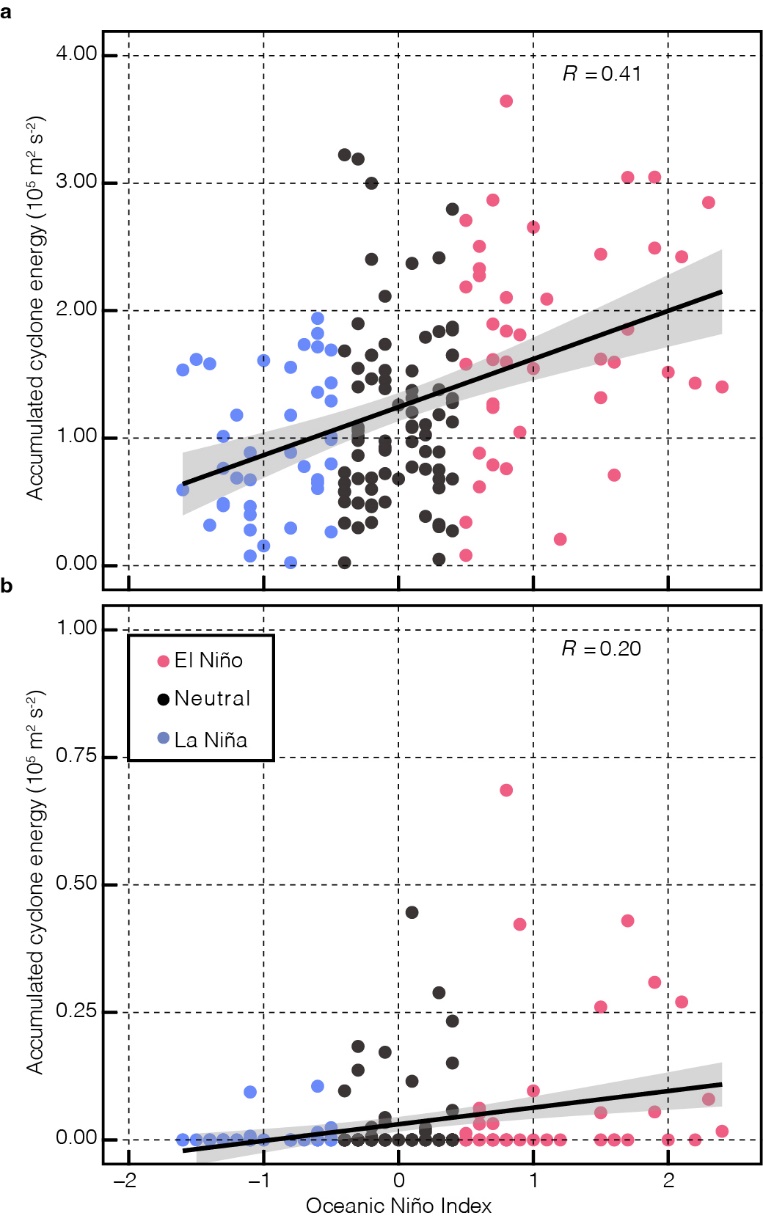
**

**Supplementary Fig. S9** | **The accumulated cyclone energy** **increases during El Niño.** **a** The tropical western north Pacific (dashed box in Fig. 3). **b** The central-north Pacific (solid box in Fig. 3).

**Supplementary Table S1 |** El Niño and La Niña cases.

| El Niño cases | | La Niña cases | |
| --- | --- | --- | --- |
| 1982 JASO | 1986 SO | 1983 SO | 1984 O |
| 1987 JASO | 1991 JASO | 1985 JA | 1988 JASO |
| **1994 SO** | 1997 JASO | 1995 ASO | 1998 JASO |
| **2002 JASO** | **2004 JASO*** | 1999 JASO | 2000 JASO |
| 2006 SO | **2009 JASO** | 2007 JASO | 2010 JASO |
| 2014 O  2018 SO | 2015 JASO | 2011 JASO  2017 O | 2016 ASO |
| 41 months | | 40 months | |

* The bold years and months represent CP El Niño cases, such as 2004 JASO.

**Supplementary Table S2 |** Suite of HighResMIP models analyzed in this study.

|  | Model Name | Ensemble Member | Atmos Nominal Resolution | Ocean Nominal Resolution |
| --- | --- | --- | --- | --- |
| 1 | CNRM-CM6-1 | r1i1p1f2 | 250 km | 100 km |
| 2 | EC-Earth3P | r1i1p2f1 | 100 km | 100 km |
| 3 | EC-Earth3P-HR | r1i1p2f1 | 50 km | 25 km |
| 4 | ECMWF-IFS-HR | r1i1p1f1 | 25 km | 25 km |
| 5 | ECMWF-IFS-LR | r1i1p1f1 | 50 km | 100 km |
| 6 | HadGEM3-GC31-HM | r1i1p1f1 | 50 km | 25 km |
| 7 | HadGEM3-GC31-LL | r1i1p1f1 | 250 km | 100 km |
| 8 | HadGEM3-GC31-MM | r1i1p1f1 | 100 km | 25 km |
| 9 | MPI-ESM1-2-HR | r1i1p1f1 | 100 km | 50 km |
| 10 | MPI-ESM1-2-XR | r1i1p1f1 | 50 km | 50 km |
